# Spatial Physics-Informed Neural Reconstruction for Ultrasound Travel-Time Tomography

**DOI:** 10.3390/s26217042

**Published:** 2026-11-07

**Authors:** Hadassah Hefter, Michael Berman

**Affiliations:** Department of Computer Science, Jerusalem Multidisciplinary College, Jerusalem 9101001, Israel; hadassahhe@edu.jmc.ac.il

**Keywords:** ultrasound tomography, travel-time tomography, speed of sound, physics-informed learning, inverse problems, autoencoder, time-of-flight extraction

## Abstract

Ultrasound computed tomography provides speed-of-sound (SoS) images that complement conventional pulse–echo ultrasound, but estimating spatial acoustic structure from transmission data remains computationally demanding. We investigate a physics-informed learned reconstruction trained exclusively on synthetic SoS fields and Eikonal-calculated travel times. Diverse synthetic structures supply paired training data at scale, enabling learning without experimentally labeled SoS images. A supervised inverse, a latent image prior, and physics-conditioned corrections produce an image that can subsequently be refined through the Eikonal equation. Evaluation on held-out synthetic fields shows improved reconstruction relative to the supervised initial estimate, with further gains from post-inference physical refinement. Applied to ToFs extracted from an in vivo breast acquisition, the fixed learned models recover heterogeneous internal SoS structure, and refinement improves correspondence with an independent full-waveform-inversion reconstruction. Controlled analyses characterize the contributions of spatial conditioning and the dependence of reconstruction on timing, support, and numerical sampling. These findings demonstrate that synthetic structures can provide useful prior information for ultrasound travel-time reconstruction and establish the feasibility of combining rapid learned inference with image-specific physical refinement.

## 1. Introduction

Ultrasound computed tomography (USCT) is a transmission-imaging approach for estimating spatially varying acoustic properties from measurements acquired around an object. In contrast to conventional pulse–echo ultrasound, which primarily depicts interfaces through reflected echoes, transmission tomography can provide quantitative maps of properties such as the speed of sound (SoS). Quantitative SoS imaging has long been investigated for breast imaging because acoustic-property contrast provides information complementary to conventional ultrasound while preserving the non-ionizing character of the modality. Early prototype studies demonstrated the feasibility of tomographic breast imaging and quantitative sound-speed estimation, and subsequent transmission systems characterized the achievable quantitative imaging performance [1,2,3].

Recovering SoS from transmission measurements is an inverse wave-propagation problem. The measured radio-frequency (RF) signals are influenced by refraction, diffraction, scattering, attenuation, and the spatial distribution of acoustic properties. Full-waveform inversion (FWI) addresses this problem by comparing measured and calculated waveforms under a wave-equation model and iteratively updating the medium parameters. Such methods can achieve high spatial resolution, but their computational cost is substantial because repeated forward and adjoint wave-equation solutions are required, and their convergence can depend strongly on initialization and on the fidelity of the assumed physical model [4,5,6,7,8,9,10].

Travel-time tomography replaces the complete waveform by estimates of first-arrival time of flight (ToF) between transmitter and receiver locations. The resulting data are substantially more compact than the RF waveforms and retain a direct relationship to SoS. Ray-based formulations commonly approximate propagation along straight or bent paths; nonlinear bent-ray inversion has been developed for circular breast-tomography systems, and in vivo studies have demonstrated improved tissue and lesion depiction when refraction is incorporated [2,11,12]. In heterogeneous media, the propagation paths themselves depend on the unknown SoS distribution, making the inverse problem nonlinear. Under a high-frequency approximation, first-arrival propagation can be described by the Eikonal equation, and numerical methods such as fast marching, ordered-upwind, finite-difference, and fast-sweeping schemes provide efficient means of calculating the corresponding travel-time fields [13,14,15,16].

Ring arrays sample many propagation directions. An acquisition-validity criterion retains reliable through-transmission channels and removes near-transmitter, glancing, weak, or otherwise unreliable measurements. The resulting incomplete path coverage defines a two-dimensional limited-view tomography problem. Reconstruction quality therefore depends on both the propagation model and the spatial information carried by the retained paths.

Learned reconstruction can transfer much of the computation to offline training [17,18,19]. Iterative, primal–dual, variational, and model-based networks combine trainable reconstruction components with information from a forward model [20,21,22,23,24]. In USCT, learned SoS reconstruction has incorporated a Tikhonov pseudoinverse as an analytical prior [25], and image-to-image learning has combined travel-time and reflection-tomography inputs for breast SoS estimation [26]. Learned FWI has also been investigated for USCT [27]; Lozenski et al. further studied neural correction of errors introduced by the Born approximation in the measurement domain, image domain, and both domains together [28].

The forward relation between a candidate SoS field and its predicted measurements provides a physical constraint on the learned inverse [29,30]. Differential-equation constraints were introduced in physics-informed neural networks and are now used in a broad range of forward and inverse problems [31,32]. In USCT, a neural surrogate trained on synthetic wave-propagation data has been incorporated into FWI and transferred to in vivo breast and musculoskeletal acquisitions [33].

We investigate a physics-informed neural reconstruction method for two-dimensional ultrasound travel-time tomography. Synthetic supervision supplies paired SoS fields and Eikonal-computed travel times at the scale required for training all learnable components. The synthetic acquisition is constructed from the transducer coordinates, channel organization, and retained-channel convention of a measured ring-array acquisition, establishing a common input representation for calculated and RF-derived ToFs. During application to measured data, the trained networks remain fixed, and the reconstructed SoS image undergoes bounded Eikonal-based physical refinement. The FWI result reported for the same acquisition by Ali et al. [6,34] is introduced after reconstruction as an independent retrospective comparator.

The varied synthetic structures provide training examples of how heterogeneous SoS distributions affect propagation, supporting reconstruction from experimental RF-derived ToFs without experimentally labeled SoS images. We evaluate the fixed learned components on the complete reserved synthetic test split, compare them with regularized bent-ray inversion initialized from homogeneous water on a predefined subset, and report post-inference refinement separately.

## 2. Physical and Data Model

The system described by Ali et al. acquires transmission data with a circular ring of transducer elements and reconstructs a cross-sectional slice through the ring. We accordingly formulate a two-dimensional problem in which the image represents the SoS distribution inside the circular array and water surrounds the imaged object. The same imaging plane defines the synthetic training domain.

Although the transducers surround the imaging region, the acquisition protocol of Ali et al. retains only a subset of receiver channels for each active transmitter [6]. Receivers near the transmitter provide less accurate measurements and are excluded, while receivers on the opposing portion of the ring provide the principal through-transmission data. This channel selection leaves incomplete angular and path coverage; the problem addressed here is therefore a two-dimensional limited-view travel-time tomography problem.

The directly measured quantities are the emitter–receiver RF waveforms. A scalar time of flight (ToF) is extracted from each retained waveform and arranged according to the transmitter–receiver geometry of the measured acquisition. The resulting ToF array, organized by transmitter and receiver, together with a validity mask identifying usable channels, forms the input to the reconstruction.

Under the high-frequency approximation, first-arrival propagation is described by the Eikonal equation [35,36]:

(1) $$\left|\nabla \tau_{e}\left(x\right)\right|=\frac{1}{c(x)},\;\tau_{e}\left(x_{e}\right)=0,$$

where c(x) is the local SoS, $\tau_{e}\left(x\right)$ is the first-arrival travel-time field for transmitter *e*, and $x_{e}$ is its location. This relation provides the physical basis for calculating synthetic travel times and for testing consistency between a reconstructed SoS image and the measured ToFs. The inverse problem is nonlinear because both the travel times and the curved propagation paths depend on the SoS distribution.

The recorded transducer coordinates of Ali et al. [6,34] are recentered to the image frame and ordered once by polar angle. Synthetic forward calculations, measured ToF arrays, and physical refinement use this common coordinate frame and channel indexing.

### 2.1. Synthetic SoS Ensemble

Each synthetic sample contains a heterogeneous two-dimensional SoS field within the circular imaging region, surrounded by water. A randomly generated circular or elliptical support envelope defines the region occupied by the synthetic structures. Its size and center vary between samples, and elliptical envelopes also vary in axis lengths and orientation. This variation exposes the learned models to objects with different spatial extents, positions, and boundary shapes. The generator produces varied spatial distributions of SoS without assigning tissue labels.

The ensemble combines randomly positioned and oriented structures with varied SoS contrast and overlap, providing paired acoustic fields and travel times for synthetic supervision. The SoS range, shape scales, and support distributions are specified in Appendix A.

The synthetic dataset is divided into separate training, validation, and test subsets. Training samples are used to optimize the learnable components, and validation samples are used to select the retained checkpoints. The test subset is reserved for evaluation after training and model selection. Dataset dimensions, acquisition configuration, and partitioning are detailed in Appendix A. The synthetic reconstruction procedure is described in Appendix B.

### 2.2. Synthetic Computed Travel Times

For each synthetic SoS field, first-arrival travel times are computed using the forward propagation model summarized by Equation (1). In this study, the synthetic first-arrival travel-time fields are computed with a fast-marching Eikonal solver, using the local SoS map as the propagation-speed field and the physical grid spacing in both spatial directions. For each transmitter, the solver produces a travel-time map from which the arrival times at the receiver locations are sampled to form the corresponding transmitter–receiver ToF data. The recorded transmitter and receiver coordinates, angular channel ordering, and validity convention of the measured acquisition are used unchanged to define the synthetic acquisition. The same channel selection is imposed when synthetic travel times are assembled. Eikonal-computed synthetic ToFs and RF-derived measured ToFs therefore use the same retained transmitter–receiver geometry and are supplied to the same reconstruction model.

### 2.3. Measured RF Acquisition

The measured breast case analyzed here is the Malignancy.mat in vivo dataset released with the implementation of Ali et al. [6,34]. It contains RF time traces for 512 emitter positions and 512 receiver positions on a 22 cm diameter circular aperture (11 cm radius), giving the 512×512 channel matrix used throughout the present work. The breast was immersed in water and positioned within the ring so that the acquisition represented a two-dimensional transverse slice. The transmitted pulse had a nominal center frequency of 2.5 MHz, and the original in vivo channel data were sampled at 12 MHz.

The 512-element representation is a spatially downsampled release of the full 1024×1024 matrix acquired by the prototype system; Ali et al. state that this reduction was made to limit the size of the distributed data files [34]. The present pipeline operates directly on the released 512-emitter, 512-receiver dataset while preserving the supplied circular geometry and angular ordering. Consistent with the through-transmission protocol of Ali et al., the opposing 75% of the receive aperture is retained; the validity criterion removes 25% of the receivers nearest to each emitter.

Measured RF waveforms are converted to first-arrival ToF estimates using geometry-guided envelope selection and local template matching. Appendix C gives the processing and timing-alignment procedure.

The channel-validity mask retains RF channels that yield reliable through-transmission ToF estimates. Near-transmitter channels, glancing paths, weak arrivals, and non-finite selections receive zero validity weight. Finite placeholder values complete the numerical array supplied to the neural networks and permit consistent display of the ToF matrix, while the mask alone determines which entries contribute to physics residuals and losses.

ToF arrays place receivers along the horizontal axis and emitters along the vertical axis.

Before reconstruction, a timing alignment compensates systematic acquisition delays using channel timing information and synthetic ToF statistics. The procedure is described in Appendix C.

**Figure 1:**
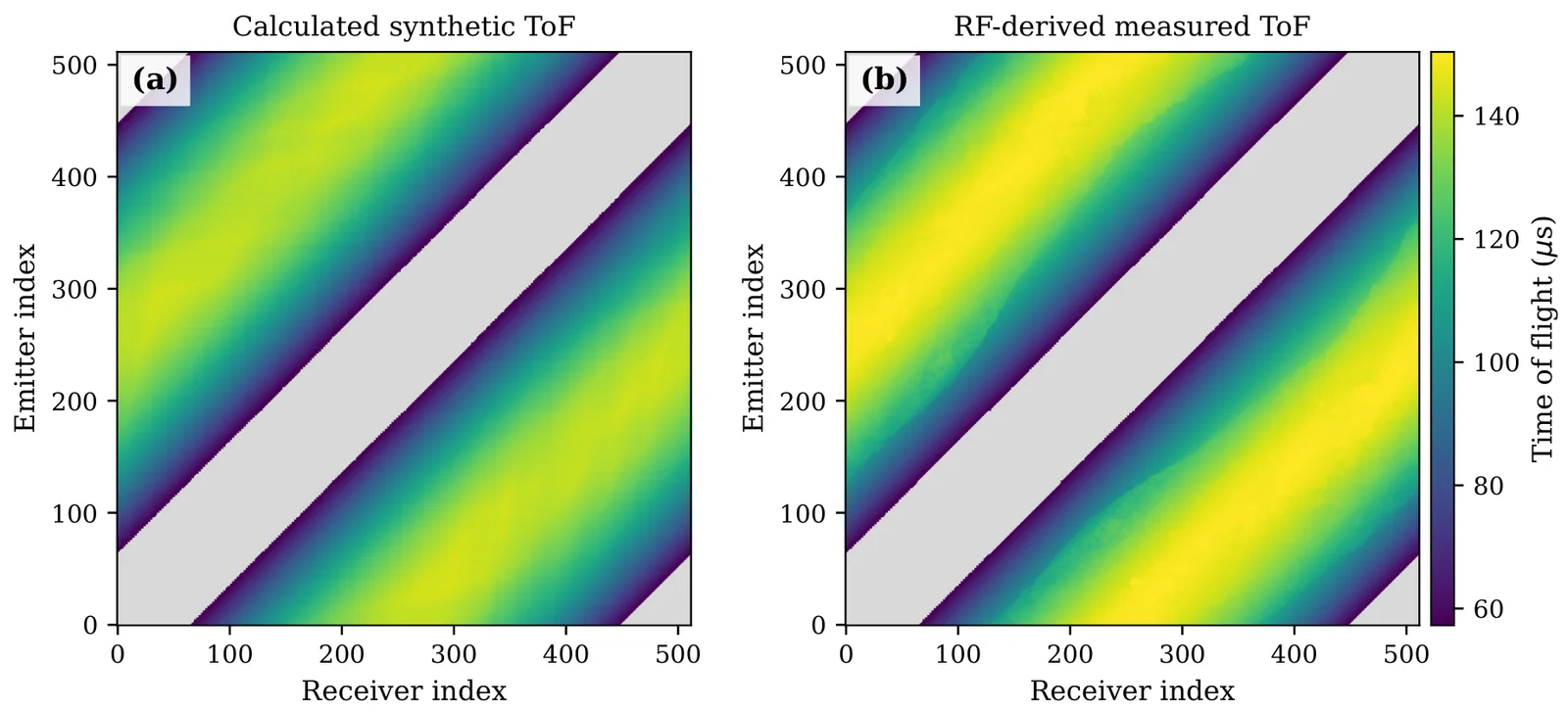
Synthetic and measured-data time-of-flight arrays organized according to the transmitter–receiver convention of the measured acquisition of Ali et al. (**a**) Calculated ToFs for the independently held-out synthetic test sample used in the reconstruction analysis. (**b**) RF-derived ToF estimates for the measured acquisition after measurement-only timing alignment. Gray regions denote transmitter–receiver channels excluded by the common limited-view validity criterion; these channels do not contribute to reconstruction losses or physics-consistency calculations. Both panels use the same scale in microseconds, with receivers shown horizontally and transmitters vertically.

In the following, RF measurements denote the recorded channel signals, RF-derived ToF estimates denote scalar arrival times selected from those signals, and calculated travel times denote forward solutions. The processed ToF array $T_{\mathrm{in}}$ and its binary channel-validity mask *M* form the reconstruction input. For emitter–receiver pair (e,r), $M_{er}=1$ denotes a retained channel, and $M_{er}=0$ denotes an excluded channel. Figure 1 shows representative calculated and RF-derived ToF arrays in this common channel organization.

## 3. Reconstruction Method

The reconstruction is formulated as an inverse travel-time problem in which measured or calculated transmitter–receiver ToFs constrain the spatial SoS field. Learning and post-inference physical refinement are separate operations. All trainable components are fitted exclusively with synthetic data generated in the geometry and channel organization of the measured acquisition; their parameters are then fixed for both synthetic testing and measured-data reconstruction.

The method comprises a supervised inverse, a latent SoS prior, a learned forward operator, and learned latent and spatial corrections. Together, they produce the learned reconstruction that initializes post-inference physical optimization.

**Figure 2:**
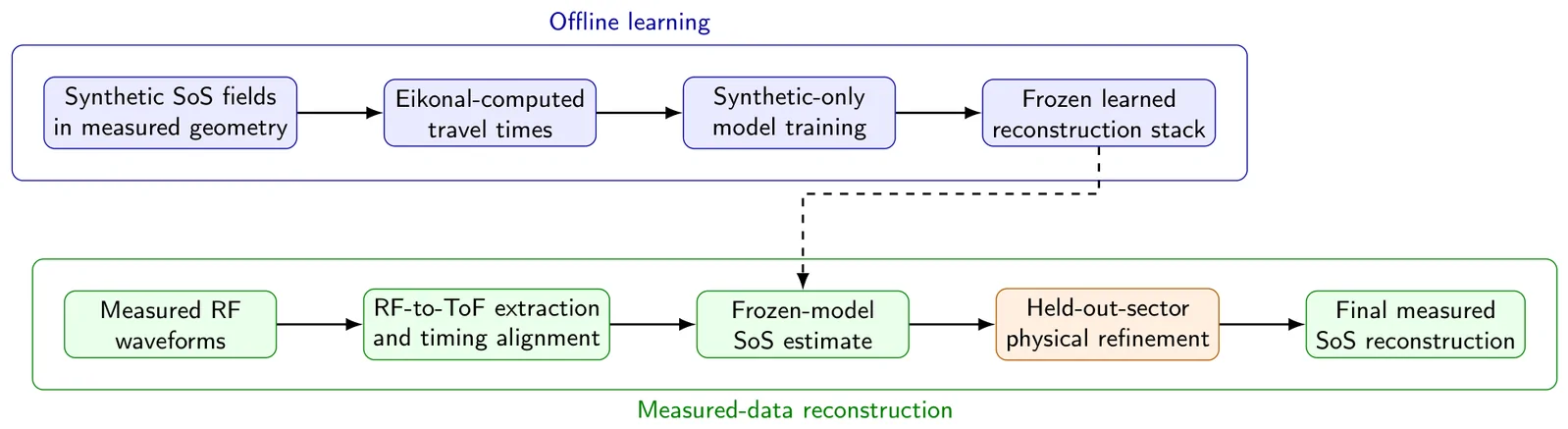
Overview of the reconstruction framework. All trainable components are learned from synthetic SoS fields and Eikonal-computed travel times generated in the measured-acquisition geometry. For measured data, RF waveforms are converted to ToFs and passed through the frozen learned stack. The resulting SoS estimate initializes a measurement-specific physical refinement based on measured ToF residuals and angularly held-out channels. Throughout this final image optimization, all learned model parameters remain fixed.

Figure 2 summarizes the learned reconstruction, synthetic training, and post-inference physical refinement.

### 3.1. Supervised Initial Reconstruction

Let $T_{\mathrm{in}}$ denote the ToF array supplied to the reconstruction after the acquisition-dependent preprocessing described in Section 2, and let *M* denote the corresponding binary channel-validity mask. For transmitter–receiver pair (e,r), $M_{er}=1$ identifies a retained channel, and $M_{er}=0$ identifies an excluded channel. The mask informs the inverse model which ToF entries are usable and restricts travel-time residuals and consistency losses to retained channels. The initial inverse model, denoted by $F_{\mathrm{inv}}$, maps these quantities to an SoS image $c_{\mathrm{init}}\left(x\right)$, where x denotes position in the two-dimensional imaging plane:

(2) $$c_{\mathrm{init}}\left(x\right)=F_{\mathrm{inv}}\;\left(T_{\mathrm{in}},M\right)\left(x\right).$$

The supervised inverse uses a fully connected encoder of the global ToF-derived input followed by a convolutional image decoder. It is trained offline and applies its fixed parameters to each new input array.

The image in Equation (2) supplies the starting point for the latent-prior correction. The inverse-model architecture is described in Appendix A.

### 3.2. Latent-Prior Physics Refinement

The initial image is next constrained by a learned prior on plausible SoS distributions. An autoencoder is trained independently on the synthetic SoS fields; after training, its decoder represents a low-dimensional family of SoS images supported by the synthetic training distribution [37,38]. The refinement combines this prior with $c_{\mathrm{init}}$, the input ToFs, and physics-derived evidence to obtain the latent-prior image $c_{\mathrm{prior}}\left(x\right)$.

The latent correction uses the residual defined in Equation (3) and its spatial conditioning features to update the autoencoder representation. The decoder converts the updated representation into $c_{\mathrm{prior}}$.

### 3.3. Operator-Matched Travel-Time Consistency

Let $T_{\mathrm{cal}}\left(c\right)$ denote the ToF array predicted by the learned forward operator for a candidate SoS field c(x). This operator is trained on synthetic SoS–ToF pairs to reproduce the same channel organization and numerical ToF representation used by the inverse model. For channel *i*, let $T_{\mathrm{in},i}$ and $T_{\mathrm{cal},i}\left(c\right)$ denote the input and predicted ToFs, respectively, and let $M_{i}=1$ for a valid channel and $M_{i}=0$ otherwise. The travel-time residual is then

(3) $$r_{i}\left(c\right)=M_{i}\;\left[T_{\mathrm{in},i}-T_{\mathrm{cal},i}\left(c\right)\right].$$

The Eikonal solver generates the synthetic first-arrival data, whereas the learned operator provides a differentiable approximation to the resulting ToF representation after acquisition-specific channel organization and preprocessing.

### 3.4. Spatial-Residual Physics Refinement

A channel residual such as $r_{i}$ acquires spatial localization through a fixed linearized straight-ray travel-time adjoint. The global map combines all valid channels, while maps formed from angular subsets preserve the direction from which the residual evidence arises. These maps associate differences between input and predicted ToFs with image locations and supply features to the correction networks during training and inference. The adjoint is a fixed mathematical operator. Its linearized sensitivity relation is given in Appendix A.

The spatial refinement receives $c_{\mathrm{prior}}$, the global and direction-resolved residual maps, and features passed from the earlier learned components. It predicts an image correction Δc(x) for which its magnitude at each pixel is constrained by a prescribed maximum, $\Delta c_{\max}>0$, expressed in the same SoS units as the image. Thus, $\left|\Delta c\left(x\right)\right|\leq \Delta c_{\max}$. During supervised synthetic training, the desired correction is the difference between the known synthetic SoS field and $c_{\mathrm{prior}}$. The final learned reconstruction is therefore

(4) $$c_{\mathrm{rec}}\left(x\right)=c_{\mathrm{prior}}\left(x\right)+\Delta c\left(x\right).$$

The construction is related to learned residual and model-based methods in which data-consistency information guides an image update [20,21,22,23,39,40]. Appendix A describes the spatial features and learned correction.

### 3.5. Synthetic Training and Model Selection

Training proceeds sequentially: The supervised inverse, autoencoder, and forward operator are fitted first; their parameters are then fixed while the latent correction is trained. With these components fixed, the spatial reconstructor is trained using Equation (4). Acquisition-like timing perturbations and channel omissions are included during correction training to promote robustness.

The networks are trained offline using AdamW, an adaptive gradient-based optimizer with weight decay, with checkpoints selected on a separate validation set. All learned parameters remain fixed during reconstruction of new inputs. Appendix A specifies the architectures, optimization safeguards, and recorded training times.

### 3.6. Frozen-Model Reconstruction and Physical Refinement

The learned reconstruction $c_{\mathrm{rec}}$ of Equation (4) supplies the initial field for a separate bounded physical optimization. Refined synthetic cases are constrained by their calculated ToFs, whereas the measured case is constrained by ToFs extracted from the RF waveforms.

Physical refinement adjusts the SoS image while penalties limit departure from the learned reconstruction and suppress spatial fluctuations. Groups of valid transmitter–receiver channels are reserved for evaluating candidate corrections and excluded from fitting. An update is retained when calculated ToFs agree more closely with the input ToFs on these reserved channels and the image correction satisfies its prescribed bounds. A rejected update stops refinement and preserves the current image. These criteria assess agreement with travel-time data; anatomical accuracy is evaluated separately using the reference images.

For measured data, the support, objective, stopping rule, acceptance criterion, and final aggregation are determined from the learned image and RF-derived ToFs. Appendix C defines the objective and describes the bounded parameterization, two-resolution procedure, and acceptance conditions. Appendix B describes the synthetic procedure.

## 4. Evaluation Framework

Evaluation compares the complete frozen learned reconstruction and the final Eikonal-refined image with the corresponding reference SoS field. The synthetic reference is the known generating field. For the measured acquisition, the FWI-derived reference reconstruction is the SoS image obtained from the same RF dataset using the MATLAB waveform-inversion implementation associated with Ali et al. [6,34]. It is introduced after reconstruction for retrospective comparison; consistency with the RF-derived ToFs is assessed separately.

### 4.1. Evaluation Quantities

The frozen physics-informed and final Eikonal-refined reconstructions are evaluated using RMSE, normalized RMSE (NRMSE), mean absolute difference (MAD), and pixelwise Pearson correlation. Their definitions are given in Appendix B, and they apply to both synthetic and measured-data evaluation. NRMSE quantifies the magnitude of the pixelwise reconstruction discrepancy on a scale normalized by the SoS variation in the reference image; smaller values indicate closer quantitative agreement. The Pearson correlation coefficient quantifies the correspondence of spatial SoS variation between the reconstruction and reference; larger positive values indicate that high- and low-SoS regions vary together across the image. Correlation is insensitive to a uniform offset and to positive multiplicative scaling, so it complements NRMSE, which remains sensitive to errors in absolute SoS level and contrast magnitude. For the synthetic test sample, $c_{\mathrm{ref}}=c_{\mathrm{true}}$, so RMSE, NRMSE, and MAD directly quantify reconstruction accuracy. For the measured acquisition, $c_{\mathrm{ref}}=c_{\mathrm{FWI}}$, which is the independently obtained FWI reconstruction, and NRMSE provides a normalized measure of the extent of agreement with that reconstruction.

These image-space measures are complemented by a travel-time consistency assessment during physical refinement: Travel times calculated from the candidate SoS image are compared with the synthetic ToFs or RF-derived ToF estimates on retained channels. For the measured breast case, image-space comparison is evaluated within an estimated breast region defined by S(x)>0.5, where *S* is a spatial support weight between zero and one. This support is derived from the frozen learned reconstruction, the water SoS, and the acquisition geometry. It represents an acquisition-centered circular region with a tapered boundary; Appendix C describes its construction.

### 4.2. Synthetic Evaluation

The supervised, latent-prior, and complete frozen learned reconstructions are evaluated on all 2000 reserved test samples, disjoint from training and validation. A predefined subset of 100 test samples is used for the comparison with regularized bent-ray inversion. Reference images enter evaluation after reconstruction and do not guide fitting or model selection. Post-inference refinement is evaluated separately for the illustrative reconstruction, an earlier three-sample set, and a predefined 20-sample set. Appendix B documents the original evaluation, and Appendix D presents the additional analyses.

The comparison method is regularized bent-ray inversion initialized from homogeneous water. It uses saved raw calculated ToFs, while the learned models use their processed synthetic inputs; the comparison therefore concerns complete workflows for the same generating fields. The sequential learned outputs show the change in accuracy as the retained components are applied. A separate matched removal-and-retraining comparison assesses the spatial residual inputs (Appendix D). Appendix B gives the bent-ray solver and iterate-selection details.

### 4.3. Evaluation of Reconstruction from RF-Derived ToFs

The RF waveforms are converted to ToF estimates, arranged in the acquisition-defined angular order, and aligned by the measurement-only procedure described in Appendix C. The frozen synthetic-trained model reconstructs the aligned ToF array, and the resulting image is refined by the held-out-sector optimization described in Section 3.6. The independently obtained FWI reconstruction reported by Ali et al. is introduced afterward solely for retrospective image-space comparison.

Quantitative comparison with the FWI reconstruction is interpreted as agreement between two reconstructions of the same RF acquisition. The FWI image serves as an independently reconstructed reference for this comparison.

### 4.4. Runtime

Runtime is reported separately for offline training, RF preprocessing, model initialization, learned inference, regularized bent-ray inversion, and physical refinement. Appendix A specifies the timing boundaries and hardware. CPU/GPU comparisons refer to the evaluated implementations.

## 5. Results

Reconstruction is evaluated on held-out synthetic SoS fields and on ToFs extracted from an in vivo breast acquisition. The known synthetic fields allow direct assessment of image accuracy; the measured-data reconstruction is compared with an independently obtained FWI image of the same acquisition. We report learned reconstruction, subsequent physical refinement, and computational cost separately. Appendix A, Appendix B and Appendix C give the learning procedures and supporting reconstruction analyses.

### 5.1. Synthetic Reconstruction

Across all 2000 test samples, the complete frozen learned reconstruction reduces mean RMSE from 45.09 to 33.25 m/s relative to the supervised inverse, a 26.3% reduction. Mean MAD decreases from 30.32 to 17.69 m/s, while mean correlation increases from 0.580 to 0.762. Table 1 reports mean and standard deviation for all four metrics. NRMSE measures errors relative to each reference image’s SoS range, whereas correlation measures spatial covariation and complements the absolute-error measures. The sequential component comparison is detailed in Appendix B.

On the predefined 100-sample subset, regularized bent-ray inversion achieves lower mean RMSE and NRMSE and higher correlation than the complete frozen learned reconstruction: 28.53 versus 32.36 m/s, 11.80% versus 13.38%, and 0.819 versus 0.766, respectively. The learned reconstruction achieves lower MAD, 17.07 versus 18.73 m/s. Table 2 reports all four measures and their variability. As described in Section 4, bent-ray inversion uses raw synthetic ToFs, whereas learned reconstruction uses processed ToFs.

**Figure 3:**
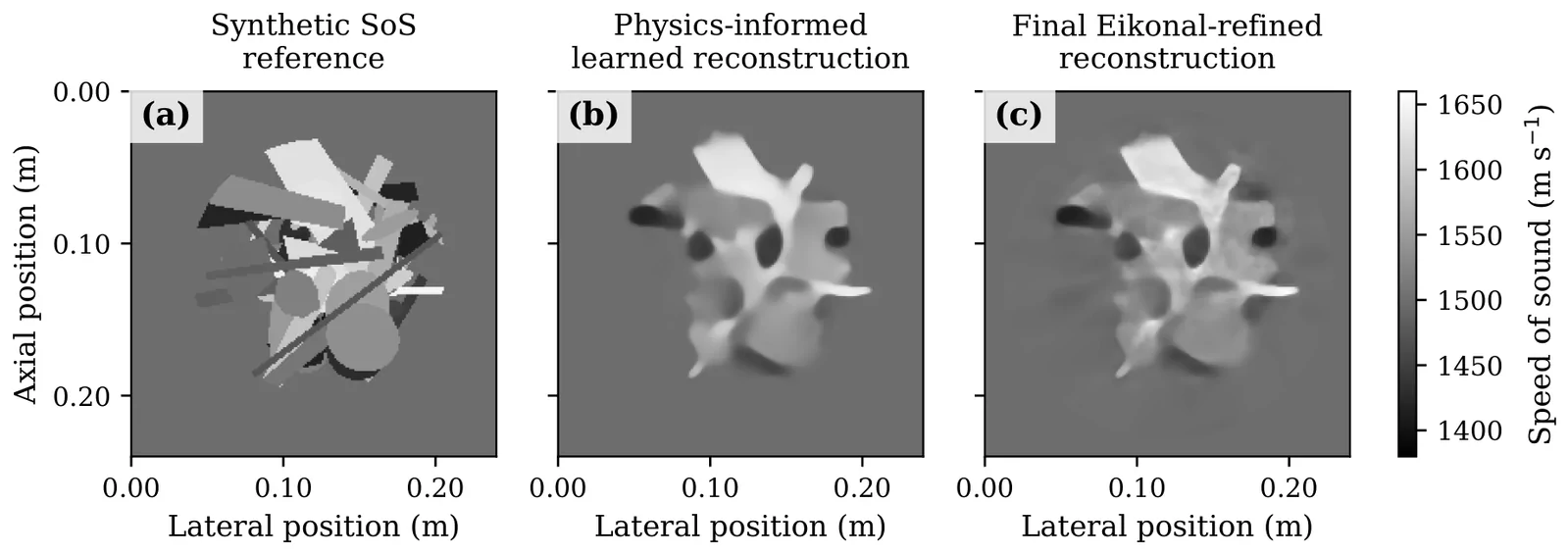
Reconstruction of an independently held-out synthetic test sample. (**a**) Known SoS field used as the evaluation reference. (**b**) Physics-informed reconstruction produced by the complete frozen learned stack before post-inference optimization. This image includes the latent SoS prior, learned forward consistency, and fixed straight-ray-adjoint conditioning. (**c**) Final reconstruction after bounded Eikonal refinement of panel (**b**). All panels use the same spatial coordinates and SoS scale. The calculated ToFs for this sample are shown in Figure 1a.

For the individual test sample shown in Figure 3, post-inference Eikonal refinement reduces NRMSE by 7.8% and increases correlation by 0.033 (Appendix B). Across the predefined 20-sample evaluation, mean NRMSE decreases by 8.4% and mean correlation increases by 4.8% relative to the entering complete learned image. Appendix D reports the paired results and variability; the earlier three-sample results remain in Appendix B.

The test sample shown in Figure 3 was selected without considering reconstruction quality. The middle and final panels show the complete learned reconstruction and its Eikonal-refined result, respectively, as described in Appendix B. The images retain similar overall structure, with changes that are subtle at the displayed scale; the quantitative comparison is given in Appendix B.

### 5.2. Reconstruction from Measured RF Data

**Figure 4:**
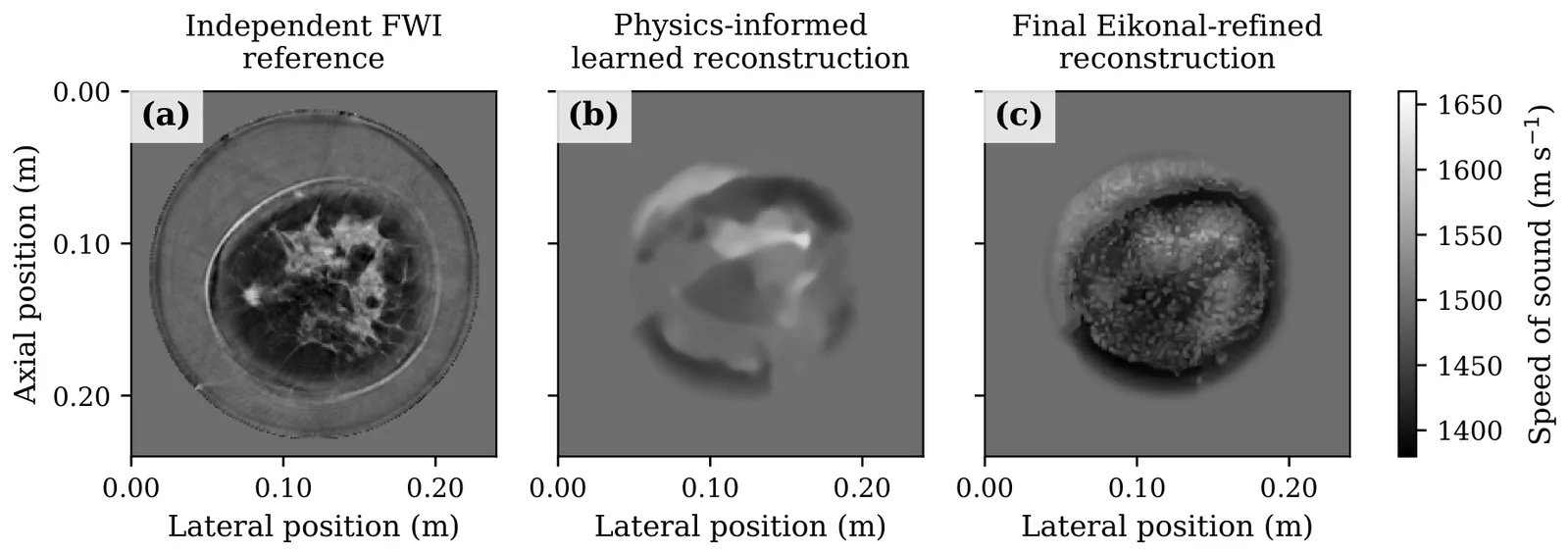
Measured breast reconstruction from the RF acquisition of Ali et al. (**a**) Independently obtained FWI reconstruction introduced after reconstruction as a retrospective evaluation reference. (**b**) Physics-informed reconstruction produced from the aligned RF-derived ToFs by the complete frozen synthetic-trained stack before post-inference optimization. (**c**) Final reconstruction after the measured-data straight-ray initialization and bounded coarse-to-fine Eikonal refinement of panel (**b**). All learned parameters remain fixed during the transition from panel (**b**) to panel (**c**). All panels use the same spatial coordinates and SoS scale.

The measured RF acquisition was converted to a ToF array and aligned using measured timing data and synthetic channel statistics. The complete frozen physics-informed learned stack produced the SoS estimate shown in the middle panel of Figure 4. Bounded physical optimization then produced the final image with all learned parameters fixed. Appendix C describes the complete measured-data workflow, including RF-to-ToF processing, frozen learned reconstruction, and physical refinement, and provides the supporting quantitative results.

Within the inferred breast support, physical refinement reduced NRMSE relative to the independently obtained FWI reconstruction by 34.6% and increased pixelwise correlation by 71.4%. Here, NRMSE measures quantitative agreement on a scale set by the FWI SoS range, whereas correlation measures correspondence of spatial SoS variation between the two reconstructions.

### 5.3. Computational Characteristics

Warm learned reconstruction takes 0.276±0.010 s per test sample after 52.23 s for loading and initialization. On the matched 100-sample subset, GPU learned inference and CPU bent-ray inversion take 0.272±0.024 s and 107.63±3.68 s per sample, respectively. These timings describe the two implemented workflows with their respective CPU/GPU allocation. Additional Eikonal refinement takes 20.3±4.9 min per case across the completed 20-sample evaluation (Appendix D). Hardware, software, offline training costs, and RF preprocessing duration are reported in Appendix A.

### 5.4. Conditioning and Sensitivity Analyses

Matched removal and retraining of the spatial residual inputs produced small, metric-dependent differences under a common training budget. This focused comparison is reported in Appendix D, separately from the original complete-model evaluation.

RF-template resampling, added waveform noise, and known-delay tests characterized arrival-selection stability. Reconstruction tests applied controlled perturbations to processed synthetic ToFs, while measured-image tests varied the support radius, center, and taper. Appendix D reports the protocols, channel counts, and image metrics.

Finer forward grids reduced numerical ToF error in homogeneous and heterogeneous test fields. Repeating physical reconstruction at three grid sizes showed different image-accuracy trends for the two synthetic samples and the measured acquisition. The complete grid analysis is reported in Appendix D.

## 6. Discussion

Across the full synthetic test set, the learned reconstruction improves on the supervised inverse, with the largest change produced by the latent-prior image and a smaller improvement from the spatial correction. On the matched 100-sample subset, regularized bent-ray inversion achieves better RMSE, NRMSE, and correlation, while learned inference achieves lower MAD and shorter reconstruction times. Runtime reflects the evaluated GPU learned implementation and CPU bent-ray implementation. The matched spatial-input ablation adds a complementary view: Under the common shortened training schedule, residual maps slightly reduce RMSE, while their removal slightly improves MAD and correlation (Appendix D). The contribution of spatial conditioning is therefore small and depends on the chosen image metric in this test.

Eikonal refinement uses travel-time data to adjust the reconstructed image after learned inference. The completed 20-sample evaluation shows improved mean image accuracy and spatial correlation alongside reduced ToF differences (Appendix D). Its paired design evaluates physical refinement separately from the original 2000-sample learned-model statistics. For the measured breast acquisition, improved agreement with an independently reconstructed FWI image demonstrates application to experimental RF-derived inputs. Known synthetic fields provide a direct image-accuracy reference; experimental FWI provides an independent reconstruction for comparison.

The transfer of learned information depends on how well the synthetic acquisition and preprocessing represent the measured input. Consistent sensor geometry, channel selection, and timing conventions are therefore essential when applying the trained model to measured data. The randomized circular and elliptical synthetic supports expose the models to variation in object size, position, and shape. In the measured reconstruction, the acquisition-centered support defines the region for physical updates and evaluation.

The synthetic structures teach the model how spatial SoS variations affect travel times and provide training information for reconstruction of heterogeneous breast SoS distributions. Application to the measured acquisition demonstrates transfer from these generated fields to experimental RF-derived inputs. This feasibility result concerns the evaluated acquisition and preprocessing. Broader experimental validation would assess transfer across different patients and acquisition conditions.

The heterogeneous Eikonal equation describes first-arrival propagation in the high-frequency approximation and incorporates refraction through spatially varying SoS [36]. Diffraction, reflected or multiply scattered arrivals, and attenuation-induced waveform-amplitude changes are waveform effects beyond this first-arrival model and can influence arrival selection from RF traces. Differences from FWI therefore reflect several contributions, including propagation-model assumptions, grid sampling, retained channels, initialization, preprocessing, and regularization. The present study addresses breast soft-tissue transmission; application to bone-containing regions or transcranial imaging would require a suitable propagation model and dedicated validation.

RF arrival selection depends on waveform noise, pulse shape, and identification of the first arrival. The repeatability analysis shows that arrival-selection stability varies between channels. The fractional-sample delay test further shows that sub-sample recovery depends on waveform interpolation and arrival selection. These results motivate reporting channelwise spread and upper quantiles alongside a median stability measure (Appendix D). Controlled processed-ToF perturbations caused small changes in mean learned-image metrics over the tested synthetic set (Appendix D). The waveform tests describe picker behavior, and the synthetic tests describe reconstruction sensitivity to the specified input errors.

The support defines the region where physical image corrections are applied, with a tapered boundary that brings the correction gradually to zero. Varying its radius, center, and taper changed FWI agreement within a relatively narrow range for the fixed entering measured image. Radius changes produced larger RMS image changes than the tested center shifts (Appendix D). Keeping the evaluation region fixed separates these changes in reconstruction from changes in the pixels used to calculate agreement.

The synthetic ensemble can be expanded without obtaining additional labeled experimental images. The present dataset contains 20,000 realizations with 45–120 structures per realization. Increasing the number of realizations would sample more spatial configurations, while varying structure counts, contrasts, scales, overlaps, and boundary shapes would broaden the image prior. These changes may improve reconstruction of finer features and transfer to measured data; their effects should be quantified on reserved test data.

The reconstruction grid has approximately 1.206 mm spacing, compared with 0.3 mm for the FWI reference. The coarser grid reduces memory and repeated forward-solution cost, and the FWI image is resampled to it for the principal comparison. Forward-grid tests show decreasing ToF error as sampling is refined. In the reconstruction-grid tests, the 200-grid result has the lowest RMSE for both synthetic samples, while the 300-grid measured reconstruction has the closest agreement with the FWI reference (Appendix D). These different trends distinguish numerical propagation accuracy from image recovery: the reconstructed structure also depends on the entering image, data representation, and regularized correction.

The use of learned information in ultrasound tomography also extends to waveform-based reconstruction. Lozenski et al. [27,28] studied learned reconstruction and correction of simplified physics in numerical breast phantoms, while Zeng et al. [33] incorporated synthetic-trained neural wave physics into iterative FWI for in vivo imaging. The travel-time representation used here offers a compact input for learned inference, with Eikonal optimization providing a subsequent physical correction. Appendix E compares the data representations and reconstruction roles in these approaches.

## 7. Conclusions

Diverse synthetic structures provide useful training information for reconstructing heterogeneous SoS fields from calculated and RF-derived travel times. The learned reconstruction improves on its supervised initial estimate and provides rapid inference after training. Subsequent Eikonal refinement improves synthetic image accuracy and agreement with an independent FWI reconstruction for the measured breast acquisition. The complementary accuracy characteristics of learned and iterative reconstruction, together with the conditioning and sensitivity analyses, clarify the role of learned priors and physical refinement in this approach. The findings establish feasibility and support evaluation across further experimental acquisitions.

## Figures and Tables

**Table 1:** Image accuracy on the complete 2000-sample synthetic test set (mean ± standard deviation). NRMSE is a percentage of each reference field’s SoS range. These results exclude post-inference Eikonal optimization.

Reconstruction	RMSE (m/s)	NRMSE (%)	MAD (m/s)	Correlation
Supervised component	45.09±6.30	18.64±2.50	30.32±5.03	0.580±0.063
Latent-prior	33.62±6.12	13.89±2.44	18.38±5.24	0.754±0.046
Complete learned	33.25±6.04	13.74±2.41	17.69±5.12	0.762±0.045

**Table 2:** Accuracy on the same predefined 100-sample subset (mean ± standard deviation). The learned workflow uses processed synthetic ToFs; regularized bent-ray inversion uses the saved raw Eikonal ToFs.

Reconstruction	RMSE (m/s)	NRMSE (%)	MAD (m/s)	Correlation
Supervised component	44.21±6.22	18.30±2.41	29.78±4.90	0.582±0.060
Latent-prior	32.72±5.68	13.53±2.22	17.75±4.74	0.758±0.040
Complete learned	32.36±5.60	13.38±2.19	17.07±4.62	0.766±0.039
Bent-ray inversion	28.53±4.20	11.80±1.62	18.73±3.29	0.819±0.036

## Data Availability

The measured RF data and waveform-inversion implementation are available from the public resources cited in the manuscript. The reconstruction software and documented configuration are available in the public GitHub repository: https://github.com/michaelberman211046/ust-spatial-physics (accessed on 6 October 2026). The public repository also includes the additional-analysis implementation and instructions for reproducing the reported results. The original source-code release v1.0.0 is archived in Zenodo (DOI: 10.5281/zenodo.23262438). Trained-model checkpoints and generated data products are not distributed; they can be reproduced using the documented synthetic-data generation and training workflow and publicly available acquisition inputs.

## Abbreviations

The following abbreviations are used in this manuscript:

FWI	Full-waveform inversion
RF	Radio frequency
SoS	Speed of sound
ToF	Time of flight
USCT	Ultrasound computed tomography

## Appendix A. Synthetic Learning and Model Construction

All learned components are fitted before reconstruction is evaluated. Parameter updates use exclusively synthetic SoS fields and their calculated ToFs. The learning sequence comprises the supervised initial inverse, the autoencoder-based latent SoS prior, the matched forward operator, and the bounded spatial correction. The autoencoder constrains image structure; propagation physics enters through Eikonal-generated ToFs, learned forward consistency, and straight-ray adjoint residual features. Measured data are introduced after completion of this training sequence.

### Appendix A.1. Training Data and Acquisition Configuration

The synthetic generator combines ellipses, triangles, polygons, and rods, with randomized location, orientation, contrast, and overlap. Each realization contains 45–120 generated structures. Structure SoS values are sampled over 1400–1650 m/s, with water at 1500 m/s. The sampled shape-axis lengths are approximately 4.8–28.9 mm on the reconstruction grid. Overlapping structures and the support boundary determine the final shapes in each SoS field.

The synthetic support envelope varies independently between samples. Its radius ranges from 65% to 95% of the array radius, with center displacements of up to 5% of that radius along either image axis. Circular and randomly oriented elliptical envelopes vary the object shape. The generated structures are confined to the envelope, with water outside it. These envelopes describe the training objects; they are distinct from the fixed imaging-domain mask and the support estimated for measured-data refinement.

The synthetic dataset contains 20,000 SoS realizations generated on a 200×200 grid spanning 0.24×0.24 m. The released Ali et al. in vivo dataset used in this work provides 512 emitter and 512 receiver positions on the 22 cm diameter ring. Those coordinates are centered in the image frame and ordered by polar angle once; the same representation and ordering are used for synthetic data and for measured RF-derived ToFs. A validity mask identifies transmitter–receiver channels that participate in the reconstruction.

The disjoint partition contains 16,000 training, 2000 validation, and 2000 test samples. Known test SoS fields are used for image metrics after reconstruction; they do not enter network fitting or selection of bent-ray iterates.

Synthetic SoS values span 1400–1650 m/s. The complete reconstruction pipeline uses fixed image-normalization bounds of 1380–1660 m/s.

The synthetic input ToFs are processed from the raw Eikonal solutions. A sample-dependent two-region circular background is used to reference the raw travel-time residual to homogeneous water: The configured inner radius is 55–70 mm, the outer-background SoS is 1460–1500 m/s, and the residual is clipped at ±8 μs. Nonintersecting channels are assigned the reference value. The inverse model and learned forward operator are trained using these processed ToFs. Post-inference physical refinement instead uses the raw Eikonal-computed ToFs. The processed ToFs are also standardized numerically before network input; SoS images are normalized separately.

Three physical operations serve different purposes. The Eikonal solver described in Section 2 generates synthetic first-arrival ToFs. During both training and frozen inference, a fixed linearized straight-ray adjoint maps ToF residuals into image-domain features used by the learned reconstruction. After frozen inference, a distinct straight-ray image update initializes the measured-data correction, which is completed by the Eikonal refinement described in Appendix C.

The accompanying code and configuration files specify the network layers, training schedules, augmentation settings, numerical solvers, and reconstruction thresholds.

### Appendix A.2. Supervised Inverse and Latent Autoencoder

The supervised inverse jointly processes the ToF-derived input channels and channel-validity information using a fully connected encoder. The encoder produces a compact spatial representation, which a convolutional decoder progressively enlarges to the SoS image. Normalization and dropout, which randomly omits features during training, stabilize learning, while a bounded output maps the prediction to the prescribed SoS range. Unlike a U-Net encoder, this encoder combines information from the entire input array through fully connected layers; the later spatial correction uses convolutional connections between corresponding image scales.

An autoencoder trained on synthetic SoS images supplies a latent image prior. Its decoder represents the spatial structures present in the synthetic training distribution and constrains the latent-prior reconstruction $c_{\mathrm{prior}}$ [37,38].

The supervised inverse and autoencoder are optimized separately on the synthetic training split. The initial inverse learns the mapping from valid-channel ToFs to SoS, whereas the autoencoder learns a compact representation directly from the SoS fields.

The SoS autoencoder compresses each training image into a latent representation and reconstructs it with a convolutional decoder, thereby learning spatial structure from the synthetic ensemble. The forward operator uses convolutional layers to extract image features and predicts the full 512×512 ToF array. The latent correction predicts bounded changes to the autoencoder representation. The spatial correction combines image and residual features at multiple scales, retaining fine-scale information through connections between its encoding and decoding layers. Its bounded output limits the magnitude of the SoS correction. Table A1 gives the parameter count of each component.

**Table A1:** Learnable model sizes from the instantiated retained models. The fixed adjoint has no trainable parameters.

Component	Parameters
Supervised inverse	837,413,673
SoS autoencoder	6,430,913
Forward operator	102,484,416
Latent correction	8,994,944
Spatial correction	3,452,353
Fixed adjoint	0

### Appendix A.3. Optimization, Environment, and Recorded Cost

All five learnable components use AdamW, an adaptive gradient-based optimizer with weight decay to penalize large network weights. Gradient clipping limits the magnitude of the gradients used for parameter updates during training of the autoencoder, forward operator, and correction networks. Table A2 lists and defines the training settings. Checkpoints are selected using separate validation data. Correction training uses up to 12,000 training samples and 512 validation samples. The supervised inverse is trained against the known SoS images, with propagation physics supplied through its calculated ToF inputs. The autoencoder learns image structure; forward consistency and adjoint residual features guide the correction networks.

**Table A2:** Training settings. Batch size is the number of samples processed together; an epoch is one pass through the training samples. Epoch entries give the scheduled maxima. Weight decay penalizes large network weights.

Component	Batch	Max. Epochs	Learning Rate	Weight Decay
Supervised inverse	8	140	$2.5\times 10^{-4}$	$1.0\times 10^{-4}$
Autoencoder	8	30	$2.0\times 10^{-4}$	$1.0\times 10^{-4}$
Forward operator	8	80	$2.0\times 10^{-4}$	$1.0\times 10^{-4}$
Latent correction	2	12	$9.0\times 10^{-5}$	$1.0\times 10^{-4}$
Spatial correction	2	18	$1.2\times 10^{-4}$	$8.0\times 10^{-5}$

During correction training, timing perturbations simulate common offsets, transmitter- and receiver-dependent delays, and random channel errors. Random omission of individual channels and groups of neighboring angular channels exposes the models to incomplete input data. These perturbations broaden the training inputs; their amplitudes are training choices rather than empirical estimates of uncertainty in RF-derived arrival times.

Learned reconstruction was timed one sample at a time, including feature construction and transfer of the reconstructed image from the GPU to CPU but excluding dataset loading and output-file writing. Bent-ray inversion timings include the iterative solution. Learned reconstruction ran on the GPU, and bent-ray inversion ran on the CPU, as specified below.

The hardware configuration comprised an Intel Core Ultra 9 285 processor (24 logical processors), 64 GB RAM, and an NVIDIA RTX PRO 2000 Blackwell GPU with 16,311 MiB device memory (approximately 15.93 GiB). Learned inference ran on the GPU, whereas the bent-ray solver ran on the CPU. The software configuration comprised Python 3.13.13, PyTorch 2.11.0+cu128, NumPy 2.5.2, SciPy 1.17.1, scikit-fmm 2025.6.23, h5py 3.16.0, and Matplotlib 3.10.9.

The recorded elapsed training times are approximately 41.3 h for the supervised inverse model, 16.8 h for the SoS autoencoder, 10.5 h for the learned forward operator, 8.7 h for the latent correction network, and 14.0 h for the spatial correction network. Synthetic-data generation takes an additional 6.6 h. These durations include training and validation.

RF-to-ToF preprocessing took approximately 49.4 min for the measured acquisition. The interval includes data loading, ToF extraction, diagnostic plotting, and output saving; timing alignment and reconstruction are subsequent operations.

The external resources used here are the released Malignancy.mat RF data and transducer coordinates and the MATLAB waveform-inversion implementation and corresponding saved comparison reconstruction associated with Ali et al. [6,34]. The present Python/PyTorch workflow constructs the synthetic fields and cached ToFs; trains the inverse, autoencoder, forward operator, and corrections; processes RF-derived ToFs; and performs the reported bounded image optimization. The independent FWI image is used for evaluation after reconstruction.

### Appendix A.4. Learned Forward-Model Consistency

The learned forward operator predicts the transmitter–receiver ToFs for a candidate SoS image. Differences between these predictions and the input ToFs supply residuals to the latent and spatial correction networks during their training and during reconstruction.

### Appendix A.5. Physics-Derived Spatial Residuals

A ToF residual identifies disagreement for an emitter–receiver pair. Let channel i=(e,r) join transmitter *e* at $x_{e}$ and receiver *r* at $x_{r}$ by the fixed straight chord $L_{er}$. Let $c_{0}\left(x\right)$ be the current SoS estimate and δc(x) a small perturbation. With dℓ denoting physical arc length along that chord, the linearized travel-time perturbation is

(A1) $$\delta T_{i}≃-\int_{L_{er}}\frac{\delta c(x)}{c_{0}^{2}\left(x\right)}\;d\ell ,\;i=\left(e,r\right).$$

The adjoint of the linearized relation in Equation (A1) distributes each channel residual back along its geometric transmitter–receiver chord with the corresponding local sensitivity. The fixed straight chord constructs an image-domain conditioning feature. The heterogeneous Eikonal solution separately determines curved first-arrival propagation paths for physical refinement.

Summing the contributions from all valid channels produces a global image-domain residual map. Repeating the operation for angular subsets of transmitters and receivers preserves directional information. This fixed adjoint calculation is used to construct conditioning features both when the correction network is trained and when the frozen network reconstructs a new input. The adjoint construction is related to linearized travel-time tomography and adjoint backprojection [4,41].

### Appendix A.6. Learned Spatial Physics-Informed Correction

The spatial reconstructor learns a bounded correction to the latent-prior SoS image. Its inputs have three complementary roles: the latent-prior image supplies the current estimate of the acoustic structure; ToF-derived features retain information from the synthetic transmitter–receiver array; the spatial residual maps described in Appendix A.5 indicate where the current image is inconsistent with the travel-time data.

A convolutional encoder–decoder combines these inputs and predicts an image-domain correction Δc. Residual blocks and skip connections are used to preserve spatial information while combining features over multiple scales [42,43]. During synthetic training, the objective balances agreement with the known SoS target, consistency with the corresponding ToFs, and spatial regularity of the correction. Direction-resolved residual maps are also used to discourage corrections that are supported by only a narrow subset of propagation directions. Table A3 summarizes the conditioning inputs.

**Table A3:** Information supplied to the final spatial correction.

Information	Role in the Reconstruction
Latent-prior SoS image	Provides the current estimate of object support and acoustic contrast.
ToF-derived image features	Preserve information from the transmitter–receiver measurements after mapping to the image domain.
Global residual map	Indicates image regions associated with the overall mismatch between predicted and input ToFs.
Direction-resolved residual maps	Retain the angular origin of residual evidence instead of averaging all propagation directions together.

## Appendix B. Reconstruction and Evaluation of Synthetic Data

The original synthetic evaluation comprises the complete 2000-sample fixed-stack test, a predefined 100-sample comparison subset, the illustrative refined example and the three-sample refinement evaluation. The additional matched ablation and 20-sample refinement evaluation are reported in Appendix D.

The comparison with regularized bent-ray inversion uses 100 held-out test samples selected at random before the aggregate evaluation. Reconstruction accuracy is assessed against the same known SoS fields within a fixed circular region of radius 0.1091 m.

### Appendix B.1. Frozen-Model Synthetic Reconstruction

The supervised inverse evaluates $F_{\mathrm{inv}}\left(T^{\mathrm{syn}},M\right)$ to obtain $c_{\mathrm{init}}$. The fixed autoencoder, learned forward operator, adjoint residual mapping, and spatial reconstructor then produce $c_{\mathrm{prior}}$ and $c_{\mathrm{rec}}$ according to the sequence defined in Section 3. All parameters remain fixed during this evaluation.

Across the complete test set, mean RMSE decreases from 45.09 m/s for the supervised component to 33.62 m/s for the latent-prior image and 33.25 m/s for the complete learned reconstruction (Table 1).

### Appendix B.2. Post-Inference Eikonal Refinement

The learned image $c_{\mathrm{rec}}$ initializes a bounded image correction driven by $T^{\mathrm{syn}}$ on valid channels. At each iteration, the Eikonal equation is solved in the candidate SoS field and sampled at the receiver positions. The resulting calculated ToFs are compared with $T^{\mathrm{syn}}$, while an anchoring term limits departure from the entering learned image and spatial regularization suppresses unsupported oscillatory corrections. Calculated ToFs are also compared with the synthetic input ToFs on groups of valid channels excluded from fitting. A correction is retained when it improves this agreement and satisfies the prescribed image-update bounds; otherwise, the current image is retained. Optimization, stopping, and acceptance are determined entirely from the calculated ToFs and the entering reconstruction, with $c_{\mathrm{true}}$ introduced afterward for evaluation.

The Adam gradient-based optimizer estimates bounded image corrections using the fitting channels. For each correction, calculated ToFs are compared with the synthetic input ToFs on channels excluded from fitting. Accepted corrections are combined, and the resulting update must also satisfy the ToF and image-update criteria. Rejection stops refinement while preserving the current image, including earlier accepted corrections. The corresponding measured-data procedure, including its straight-ray initialization, tapered support, and common timing correction, is described in Appendix C.

The optimization is performed first on a coarser correction representation and then on a finer representation initialized from the accepted coarse result. The coarse representation permits broad image changes; the finer representation permits more localized corrections. The final accepted field is denoted by $c_{\mathrm{phys}}^{\mathrm{syn}}$.

### Appendix B.3. Quantitative Evaluation of the Held-Out Synthetic Reconstruction

The same four image-space quantities are used to compare the complete frozen physics-informed reconstruction with the final Eikonal-refined reconstruction in the synthetic and measured settings. Let Ω be the evaluation support, $N_{\Omega }=\left|\Omega \right|$; c(x) a reconstructed SoS field; and $c_{\mathrm{ref}}\left(x\right)$ the corresponding reference image. Define the reference SoS range over Ω as $R_{\mathrm{ref}}=\max_{\Omega }c_{\mathrm{ref}}-\min_{\Omega }c_{\mathrm{ref}}$. The root-mean-square error (RMSE), normalized RMSE (NRMSE), mean absolute difference (MAD), and pixelwise Pearson correlation are

(A2) $$\begin{array}{cc} E_{\mathrm{RMSE}}\left(c,c_{\mathrm{ref}}\right)\; & ={\left\{\frac{1}{N_{\Omega }}\sum_{x\in \Omega }{\left[c\left(x\right)-c_{\mathrm{ref}}\left(x\right)\right]}^{2}\right\}}^{1/2},\\ E_{\mathrm{NRMSE}}\left(c,c_{\mathrm{ref}}\right)\; & =100\;\frac{E_{\mathrm{RMSE}}\left(c,c_{\mathrm{ref}}\right)}{R_{\mathrm{ref}}},\\ E_{\mathrm{MAD}}\left(c,c_{\mathrm{ref}}\right)\; & =\frac{1}{N_{\Omega }}\sum_{x\in \Omega }\left|c\left(x\right)-c_{\mathrm{ref}}\left(x\right)\right|,\\ \rho (c,c_{\mathrm{ref}})\; & =\frac{\sum_{x\in \Omega }\left[c\left(x\right)-\bar{c}\right]\left[c_{\mathrm{ref}}\left(x\right)-{\bar{c}}_{\mathrm{ref}}\right]}{{\left\{\sum_{x\in \Omega }{\left[c\left(x\right)-\bar{c}\right]}^{2}\sum_{x\in \Omega }{\left[c_{\mathrm{ref}}\left(x\right)-{\bar{c}}_{\mathrm{ref}}\right]}^{2}\right\}}^{1/2}}, \end{array}$$

In Equation (A2), $\bar{c}$ and ${\bar{c}}_{\mathrm{ref}}$ are the respective mean values over Ω, and NRMSE is expressed as a percentage of the reference SoS range.

Table A4 compares the complete frozen-stack reconstruction, $c_{\mathrm{rec}}$, with the final Eikonal-refined reconstruction, $c_{\mathrm{phys}}^{\mathrm{syn}}$, for the independently held-out test sample shown in Figure 3. All four measures are evaluated within the circular reconstruction support against the known SoS field, $c_{\mathrm{true}}$.

**Table A4:** Quantitative comparison of the frozen physics-informed and final Eikonal-refined reconstructions for the independently held-out synthetic test sample.

Metric	FrozenPhysics-Informed	Final Eikonal-Refined	Change
RMSE (m/s)	27.85	25.67	7.8% reduction
NRMSE (%)	11.32	10.43	7.8% reduction
Mean absolute difference (m/s)	13.13	12.90	1.7% reduction
Pixelwise correlation	0.777	0.810	+0.033 (4.3%)

For the error measures, the percentage reduction is calculated relative to the frozen physics-informed value. For correlation, the table gives both the absolute increase and the increase relative to the frozen physics-informed correlation.

### Appendix B.4. Regularized Bent-Ray Travel-Time Inversion

Regularized bent-ray inversion starts from a uniform SoS of 1500 m/s, corresponding to water. It adjusts the image to reduce differences between the raw synthetic ToFs and travel times calculated through the current SoS field. Quadratic regularization penalizes differences between neighboring image pixels and departure from the water SoS, limiting spatial fluctuations and large changes in acoustic contrast. At each reconstruction step, the Eikonal equation supplies first-arrival travel-time fields from which curved propagation paths are traced. These paths determine how small changes in each image region affect the calculated travel times. A regularized least-squares calculation then estimates an image correction. The correction is bounded, and its magnitude is reduced if necessary until the combined travel-time and regularization objective decreases. Propagation paths are recalculated after each accepted correction. To reduce computational cost, the calculation uses a uniformly spaced subset of the available transmitters and receivers and retains channels allowed by the validity mask. Selected transmitter directions are reserved for checking the reconstruction and are excluded from fitting. Among the successive reconstructions, the final image is the one with the smallest root-mean-square difference between calculated and input ToFs on these reserved channels. The known SoS field is used afterward for image-accuracy evaluation.

Table 2 reports mean and standard deviation of the image metrics on the 100-sample comparison subset, with results discussed in Section 5. Regularized bent-ray inversion achieves lower RMSE and higher correlation, whereas the complete learned reconstruction achieves lower MAD and shorter reconstruction time. All methods are evaluated against the same known fields on the same fixed support. The learned reconstruction uses processed synthetic ToFs, and bent-ray inversion uses raw Eikonal-computed ToFs, as specified in Section 4.

### Appendix B.5. Three-Sample Eikonal-Refinement Evaluation

Three additional test samples from the predefined subset were evaluated before and after coarse-to-fine refinement. Table A5 retains these earlier results. The completed 20-sample evaluation, including paired metrics and updated timing, is reported in Appendix D.

**Table A5:** Three-sample Eikonal-refinement evaluation (mean ± standard deviation). The same three cases are included before and after refinement, including cases rejecting further fine updates.

Metric	Frozen Learned	After Refinement
RMSE (m/s)	32.53±9.87	29.30±8.94
NRMSE (%)	13.56±3.90	12.21±3.51
MAD (m/s)	18.38±7.65	17.17±7.44
Correlation	0.787±0.082	0.830±0.059

## Appendix C. Reconstruction from Experimental RF Data Through Extracted ToFs

Reconstruction from measured RF data comprises ToF extraction and timing alignment, inference with the synthetic-trained model, and physical refinement. The independent FWI reconstruction supplies the comparison image after reconstruction is complete.

### Appendix C.1. RF-to-ToF Processing

The RF data and circular-array geometry are those of Ali et al. [6,34], who acquired the breast data in water immersion. For each retained transmitter–receiver waveform, the sensor separation and travel time in water define a physically plausible first-arrival interval. The signal envelope supplies an initial arrival estimate within that interval. A representative template formed from reliable measured traces refines the estimate by maximizing normalized waveform agreement over a short local window. If the template match is unreliable, the envelope estimate is retained. Channel-validity screening and subsequent timing alignment address unreliable arrival estimates and systematic timing offsets.

The extracted ToFs are arranged in the same angular transmitter–receiver order as the calculated training ToFs. A measurement-based timing alignment compensates systematic electronic delays that are absent from the propagation model. If $T_{er}^{\mathrm{RF}}$ is the extracted ToF for transmitter *e* and receiver *r*, the aligned value is

(A3) $$T_{er}^{\mathrm{align}}=T_{er}^{\mathrm{RF}}-\left(a+b_{e}+d_{r}\right),$$

where *a* is a common delay, and $b_{e}$ and $d_{r}$ are transmitter- and receiver-dependent terms. These terms are estimated from channel timing information and retained when they reduce the discrepancy on channels withheld from estimation. The aligned array in Equation (A3) and its validity mask form the input to the frozen learned model; masked entries do not contribute to physical residuals or losses.

### Appendix C.2. Frozen Learned Reconstruction

The aligned ToF array and validity mask are processed by the learned reconstruction described in Appendix A, with all network parameters fixed. The resulting SoS image initializes physical refinement.

### Appendix C.3. Measured-Data Physical Refinement

A straight-ray update adjusts the SoS image using discrepancies between RF-derived ToFs and travel times calculated by integrating the reciprocal SoS along straight transmitter–receiver paths. Coarse and fine refinement recompute Eikonal first-arrival travel times through each candidate SoS field, incorporating the effects of spatially varying SoS on propagation.

Let $c_{j}$ be the image entering refinement step *j*, δc its candidate correction, and *b* a bounded common timing term. The correction is obtained by minimizing the objective in Equation (A4):

(A4) $$\begin{array}{cc} J(\delta c,b)= & D_{\mathrm{Huber}}\;\left(T^{\mathrm{Eik}}\left(c_{j}+\delta c\right)+b,T^{\mathrm{align}}\right)\\ \; & +\lambda_{\mathrm{prior}}A\left(\delta c\right)+\lambda_{\mathrm{TV}}\mathrm{TV}\left(\delta c\right)+\lambda_{\mathrm{smooth}}R_{\mathrm{smooth}}\left(\delta c\right), \end{array}$$

where $T^{\mathrm{Eik}}$ denotes Eikonal-computed receiver arrival times, and the data term uses valid channels selected for fitting. The Huber penalty $D_{\mathrm{Huber}}$ is quadratic for small ToF residuals and linear for large residuals [44]. The anchoring penalty A limits departure from the entering image, total variation TV penalizes first spatial differences while allowing boundaries, and $R_{\mathrm{smooth}}$ suppresses short-wavelength fluctuations. The non-negative coefficients $\lambda_{\mathrm{prior}}$, $\lambda_{\mathrm{TV}}$, and $\lambda_{\mathrm{smooth}}$ set their respective weights [45,46].

The support weight S(x)∈[0,1] defines a circular region with a tapered boundary, estimated from the learned image’s contrast relative to water and constrained by the acquisition geometry. Refinement preserves the image outside this region. Within it, bounded SoS corrections have zero support-weighted mean, separating spatial changes from a bounded common timing adjustment.

Refinement uses the Adam gradient-based optimizer to estimate bounded image corrections. Among the channels retained by the acquisition-validity mask described in Section 2.3, groups of transmitter directions are temporarily withheld from fitting. Each correction is evaluated by comparing its calculated ToFs with the RF-derived ToFs on these withheld channels. Accepted corrections must reduce the median absolute ToF difference, keep the root-mean-square difference within its prescribed bound, and satisfy the image-update bounds. Corrections accepted in several fits with different withheld directions are combined by their median at each pixel; the combined correction is reduced at pixels where the individual corrections disagree. The combined correction is checked against the ToF and image-update bounds before acceptance. Rejection stops refinement and preserves the current image, including earlier accepted corrections. Selection uses the RF-derived ToFs independently of the FWI reference.

### Appendix C.4. Quantitative Evaluation of the Measured Reconstruction

The RF traces were sampled at 12 MHz, corresponding to a sample interval of approximately 0.083 μs. Arrival times were estimated using the envelope and template-matching procedure in Appendix C.1. Timing alignment subtracted common, transmitter-dependent, and receiver-dependent delay estimates before learned reconstruction. During physical refinement, an additional bounded common timing term was fitted together with the image correction. Processing settings are specified in the accompanying code repository.

Physical refinement used the support weight *S* defined in Appendix C.3. The frozen learned and final refined images were compared with the FWI reference over the same region, defined by S>0.5.

The RF-to-ToF procedure returned finite accepted estimates for more than 99.9% of channels eligible under the acquisition mask. This fraction describes the availability of ToF estimates under the picker rules; arrival-time accuracy depends on waveform quality and correct first-arrival identification, as discussed in Section 6.

Timing alignment reduced the median absolute discrepancy between measured and synthetic channel timing statistics from 2.095 to 0.0436 μs on channels withheld from alignment estimation.

The Ali et al. MATLAB FWI reference uses an 801×801 Cartesian grid spanning −0.12 to 0.12 m in each direction, with 0.3 mm spacing. It was resampled by bilinear interpolation in physical coordinates onto the 200×200 reconstruction grid over the same field of view, preserving its MATLAB display orientation. Both reconstructed images were therefore compared with the FWI reference on the same grid.

Comparison with the resampled FWI image uses the region S>0.5, comprising 12,540 pixels, with *S* defined in Appendix C.3. Table A6 reports the same RMSE, NRMSE, mean absolute difference, and pixelwise correlation measures used for the synthetic test sample, comparing the frozen physics-informed image with the final Eikonal-refined image.

**Table A6:** Quantitative comparison of the frozen physics-informed and final Eikonal-refined reconstructions for the measured breast acquisition, evaluated relative to the independently obtained FWI reconstruction.

Metric	Frozen Physics-Informed	Final Eikonal-Refined	Change
RMSE (m/s)	56.19	36.76	34.6% reduction
NRMSE (%)	22.04	14.42	34.6% reduction
Mean absolute difference (m/s)	47.88	29.79	37.8% reduction
Pixelwise correlation	0.322	0.552	+0.230 (71.4%)

The percentage reductions are calculated relative to the frozen physics-informed error measures. The correlation entry reports its absolute increase followed by the relative percentage increase.

## Appendix D. Additional Reconstruction Analyses

The analyses below examine the information supplied to the spatial correction; physical refinement across a predefined test set, RF arrival selection; and sensitivity to input timing, spatial support, and numerical grid. They use the saved training, validation, and test partition. Reference SoS fields and the independent FWI image enter image evaluation after reconstruction. RMSE and mean absolute difference (MAD) are expressed in m/s; NRMSE is RMSE divided by the reference SoS range in the evaluation region, multiplied by 100. Correlation measures pixelwise spatial covariation. The implementation and configurations are provided in the additional_analysis directory of the public software repository.

### Appendix D.1. Removal and Retraining of Spatial Residual Inputs

Two spatial correction networks were trained anew with the same 256 training samples, 64 validation samples, six training epochs, and one common random seed. The upstream inverse, autoencoder, forward operator, and latent correction remained fixed. One network received all 53 conditioning channels; the other received 14 channels after removal of the explicit global and directional spatial residual maps. Retained input weights shared their initial values across the two variants. Both variants used the same image and forward-operator losses, and the checkpoint with the lowest validation image MSE was retained. This matched experiment used clean cached inputs and omitted the nuisance and consistency augmentations of the original training schedule. Both fitted variants were then evaluated on all 2000 reserved test samples.

**Table A7:** Matched spatial-input removal and retraining, evaluated on 2000 test samples (mean ± standard deviation). Both variants use the same shortened training budget.

Inputs	RMSE	NRMSE (%)	MAD	Correlation
All conditioning inputs	33.60±6.11	13.88±2.44	18.51±5.20	0.755±0.046
Spatial residual maps removed	33.64±6.10	13.90±2.43	18.32±5.20	0.755±0.046

With the residual maps, mean RMSE was lower by 0.038 m/s, while MAD was higher by 0.193 m/s and correlation was lower by 0.000385 (Table A7). The paired 95% bootstrap interval for the RMSE difference, removed-input minus full-input, was 0.034–0.042 m/s. Resampling used 1000 draws of paired test samples and describes uncertainty conditional on these two fitted networks. The differences are small and metric-dependent under this training budget. The originally trained complete network retains the separate performance reported in Table 1.

### Appendix D.2. Expanded Synthetic Eikonal Refinement

Twenty test indices were selected from a fixed random permutation of the reserved test split before refinement. Each original frozen learned reconstruction initialized the coarse-to-fine Eikonal procedure of Appendix B. All 20 selected cases were completed and retained in the analysis, including rejected fine updates. The generating fields were used afterward for the image metrics on the same circular evaluation region of radius 0.1091 m. ToF RMS differences were computed from physical Eikonal arrivals and the saved raw calculated ToFs on the same diagnostic channels for each entering and final image.

**Table A8:** Physical refinement across all 20 selected synthetic test samples (mean ± standard deviation). The entering image is the original complete frozen learned reconstruction.

Quantity	Entering Image	Refined Image
RMSE (m/s)	31.27±5.51	28.65±4.71
NRMSE (%)	12.92±2.16	11.84±1.84
MAD (m/s)	15.73±4.29	15.44±3.86
Correlation	0.771±0.043	0.809±0.035
ToF RMS difference (μs)	0.387±0.163	0.128±0.039

Table A8 shows an 8.4% decrease in mean NRMSE and a 4.8% increase in mean correlation. The paired mean RMSE change was −2.62 m/s (95% bootstrap interval −3.02 to −2.24 m/s); the correlation change was +0.037 (interval 0.032–0.043). Mean refinement time was 20.3±4.9 min per case, excluding the initial learned inference and subsequent diagnostic calculations. These results are evaluated separately from the complete-test learned-model statistics and the earlier three-sample refinement results.

### Appendix D.3. RF-Picker Repeatability and Known-Delay Recovery

The retained envelope/template picker was reapplied to 512 acquisition-valid channels selected before waveform inspection. The pulse template was rebuilt from the full acquisition and the resulting baseline picks reproduced all comparable saved picks within 0.01 μs. Table A9 summarizes 20 repeat estimates using bootstrap resampling of the template traces. Added Gaussian noise was scaled by 0.5, 1 or 2 times each channel’s pre-arrival noise estimate, obtained as the median absolute deviation of the raw pre-arrival samples divided by 0.67449. A positive estimate was available for 505 channels; seven channels had zero pre-arrival median absolute deviation. Template-only and known-delay tests retained all 512 channels, while added-noise tests used the explicitly identified 505-channel subset. The added-noise levels supplement the noise already present in each trace.

**Table A9:** RF picking under template resampling and added waveform noise. SD is each channel’s standard deviation over 20 repeats. The last column is the RMS change from the unperturbed pick across channel–repeat pairs; all timing quantities are in μs.

Condition	Channels	Median SD	95th Percentile SD	RMS Change
Template only	512	0.0012	0.1030	0.0468
Added noise ×0.5	505	0.0013	0.1021	0.0588
Added noise ×1	505	0.0012	0.1315	0.0794
Added noise ×2	505	0.0016	0.1496	0.1137

To assess recovery of known timing increments, each raw trace was shifted by −0.25, +0.04, or +0.25 μs using interpolation on the RF time axis and then repicked with the baseline template. The recovered increment was the shifted pick minus the baseline pick; its error was evaluated against the imposed shift. Whole-sample shifts of ±0.25 μs were recovered to numerical precision (RMS increment error below $10^{-10}$ μs). For the fractional-sample shift of 0.04 μs, the mean increment error was −0.0328 μs, RMS error was 0.2679 μs, and the 95th percentile absolute error was 0.7109 μs. All 512 channels returned picks in these tests. These quantities characterize repeatability and incremental shift recovery conditional on the retained waveforms and picker, separately from absolute first-arrival accuracy.

### Appendix D.4. Sensitivity of Learned Reconstruction to ToF Perturbations

The original frozen learned reconstruction was recomputed for 20 predefined test samples after perturbing their processed synthetic ToFs. Independent Gaussian jitter had a standard deviation of 0.03, 0.08, or 0.20 μs. Common offsets of ±0.20 μs were added to every valid channel. Element-dependent errors were the sum of independent transmitter and receiver offsets, each with a standard deviation of 0.10 μs. Channel dropout excluded a random 5% of valid entries and updated the input mask. All upstream learned components and spatial features were recomputed for each perturbed input; target images and the circular image-evaluation region remained fixed. These tests concern learned inference before physical refinement; Table A10 reports their image metrics.

**Table A10:** Mean image metrics after controlled processed-ToF perturbations across the same 20 test samples. Timing parameters denote standard deviations for jitter/element errors and signed shifts for common offsets.

Condition	RMSE (m/s)	NRMSE (%)	MAD (m/s)	Correlation
Unperturbed	31.27	12.92	15.73	0.771
Jitter 0.03 μs	31.27	12.92	15.73	0.772
Jitter 0.08 μs	31.28	12.93	15.74	0.771
Jitter 0.20 μs	31.36	12.96	15.81	0.770
Common −0.20 μs	31.22	12.90	15.72	0.772
Common +0.20μs	31.36	12.96	15.78	0.770
Element offsets 0.10 μs	31.28	12.93	15.74	0.771
Channel dropout 5%	31.35	12.96	15.82	0.770

The largest mean RMSE among the tested perturbations was 31.36 m/s, compared with 31.27 m/s for the unperturbed inputs. The corresponding changes quantify sensitivity to the stated perturbations in this synthetic input representation; they complement the RF-waveform tests in Appendix D.3.

### Appendix D.5. Sensitivity to the Measured-Image Support

The measured-data Eikonal optimization was repeated with nine support weights: the nominal support, radius changes of ±5%, center shifts of ±2 mm along each coordinate axis, and taper-width factors of 0.5 and 2. The support weight *S* is defined in Appendix C. Every fit started from the same saved image after the initial straight-ray physical correction and used the same RF-derived ToFs. Image evaluation used the fixed nominal region S>0.5 for all fits; the FWI reference entered this evaluation after optimization. The tests therefore isolate the support used in subsequent Eikonal corrections while holding the entering reconstruction fixed.

**Table A11:** FWI agreement after measured-data Eikonal refinement with perturbed supports. RMS Δc is the root-mean-square image difference from the nominal-support refined image over the same fixed evaluation region. All image differences are in m/s.

Support	RMSE	NRMSE (%)	MAD	Correlation	RMS Δc
Nominal	36.76	14.42	29.79	0.552	0.00
Radius −5%	37.21	14.59	30.05	0.549	3.85
Radius +5%	36.24	14.22	29.35	0.561	3.06
Center x−2 mm	36.94	14.49	29.99	0.551	1.39
Center x+2 mm	36.63	14.37	29.58	0.554	1.52
Center y−2 mm	36.95	14.49	30.02	0.552	1.53
Center y+2 mm	36.60	14.36	29.54	0.553	1.41
Taper ×0.5	36.29	14.23	29.34	0.562	2.35
Taper ×2	37.57	14.73	30.44	0.542	3.07

Table A11 reports the resulting image metrics. Across these variants, FWI NRMSE ranged from 14.22% to 14.73%, and correlation ranged from 0.542 to 0.562. The largest RMS image change from nominal was 3.85 m/s for the 5% radius reduction. Diagnostic ToF RMS differences ranged from 1.69 to 1.78 μs after fitting a bounded common timing offset to each final image for this diagnostic calculation. These quantities assess sensitivity for the supplied acquisition and fixed entering image.

### Appendix D.6. Forward-Grid Convergence and Reconstruction-Grid Effects

Forward-grid convergence used one homogeneous water field (1500 m/s) and one analytic heterogeneous field containing two elliptical contrasts (1450 and 1580 m/s). Each field was sampled independently on grids of 100×100, 200×200 and 400×400 over the same 0.24 m square. First-arrival ToFs were calculated at the measured sensor coordinates for 16 fixed transmitters and 128 receivers, retaining 1535 acquisition-valid pairs. Analytic chord travel times provided the homogeneous reference; an 801×801 Eikonal solution provided the heterogeneous numerical reference. Table A12 reports errors including the implemented sensor-to-grid and receiver-sampling conventions.

**Table A12:** Forward ToF error under grid refinement. The homogeneous reference is analytic; the heterogeneous reference is the 801-grid numerical solution. Timing errors are in μs.

Field	Grid per Axis	RMS Error	95th Percentile Absolute Error
Homogeneous	100	1.192	2.293
Homogeneous	200	0.599	1.135
Homogeneous	400	0.274	0.522
Heterogeneous	100	1.076	2.088
Heterogeneous	200	0.505	0.977
Heterogeneous	400	0.197	0.370

The reconstruction-grid analysis reran physical optimization on 100, 200, and 300 points per axis, using the same physical domain, sensor coordinates, ToFs and correction-basis sizes. The learned entering image was resampled in physical coordinates to each grid; results were mapped back to the original 200-grid evaluation region. Resampling-only images provide controls for the effect of interpolation before optimization. The physical characteristic-tracing increment was matched across grids. All nine calculations were completed: two predetermined synthetic samples and the measured acquisition at each of the three grids (Table A13). Synthetic cases 1 and 2 correspond to saved test indices 13,013 and 14,351. Each image is evaluated against its corresponding known synthetic field or independent FWI reference on the same original 200-grid evaluation region.

**Table A13:** Reconstruction-grid results for the two synthetic cases and measured acquisition. Control RMSE uses the resampled entering image; final metrics follow fresh Eikonal optimization at the stated grid. RMSE is in m/s.

Case	Grid	Control RMSE	Final RMSE	NRMSE (%)	Correlation
Synthetic 1	100	31.84	31.08	12.70	0.771
Synthetic 1	200	32.33	29.64	12.11	0.796
Synthetic 1	300	32.13	31.35	12.81	0.769
Synthetic 2	100	17.83	17.82	7.70	0.891
Synthetic 2	200	17.92	17.27	7.47	0.897
Synthetic 2	300	17.81	17.76	7.68	0.892
Measured	100	37.01	36.17	14.19	0.562
Measured	200	39.25	36.76	14.42	0.552
Measured	300	38.29	35.72	14.01	0.574

Forward ToF errors decreased with grid refinement for both analytic fields. The 200-grid result had the lowest RMSE for both synthetic samples. For the measured acquisition, the 300-grid reconstruction achieved the lowest FWI RMSE (35.72 m/s), NRMSE (14.01%), and MAD (29.11 m/s) and the highest correlation (0.574). Relative to the 200-grid result, its FWI NRMSE decreased by 2.8% and correlation increased by 4.0%. The interpolation-only controls also vary with grid size, identifying a contribution from resampling the entering image. These tests assess forward numerical accuracy and fresh post-inference optimization at each grid; all comparisons use the original common evaluation grid and the same learned entering information.

## Appendix E. Comparison with Related Learned USCT Reconstruction Studies

The studies of Lozenski et al. [27,28] use simulated waveform data to investigate learned USCT reconstruction, either through a direct learned mapping or through learned correction of simplified propagation physics. Zeng et al. [33] trained a neural surrogate for wave propagation on synthetic data and used it within iterative FWI for in vivo breast and musculoskeletal imaging.

The present method applies a synthetic-trained inverse to measured first-arrival ToFs. Its neural-network parameters remain fixed for the measured case, and a bounded measurement-specific ray optimization subsequently refines the reconstructed SoS field.
